# Identification and monitoring of Korean medicines derived from *Cinnamomum* spp. by using ITS and DNA marker

**DOI:** 10.1007/s13258-016-0476-5

**Published:** 2016-10-20

**Authors:** Eui Jeong Doh, Jung-Hoon Kim, Seung eun Oh, Guemsan Lee

**Affiliations:** 1 Department of Herbology, College of Korean Medicine, Wonkwang University, Iksan, 54538 Republic of Korea; 2 Division of Pharmacology, School of Korean Medicine, Pusan National University, Yangsan, 50612 Republic of Korea; 3 Division of Biological Sciences, Konkuk University, Seoul, 05029 Republic of Korea; 4 Center for Metabolic Function Regulation, Wonkwang University, Iksan, 54538 Republic of Korea

**Keywords:** *Cinnamomum cassia*, ITS (Internal transcribed spacer), Cinnamomi Ramulus, Cinnamomi Cortex, Cassiae Cortex Interior

## Abstract

In this study, we identified and evaluated the genetic relationships among *Cinnamomum* plants, which are used in traditional medicine. We also attempted to monitor the distribution of traditional medicines derived from *Cinnamomum cassia* by using DNA barcoding and a species-specific DNA marker. Plants of the genus *Cinnamomum*, and in particular *C. cassia*, are commonly used as medicinal herbs in the form of Cinnamomi Ramulus, Cinnamomi Cortex, and Cassiae Cortex Interior. However, it is difficult to distinguish among different *Cinnamomum* species based on morphological features, and so to overcome this limitation, nucleotide sequences of the internal transcribed spacer (ITS) region of *Cinnamomum* DNA were determined and compared. On the basis of the discrepancy in determined ITS sequences, a 408-bp product, amplified by the primer pair CC F1/CC R3, was developed as a *C. cassia*-specific DNA marker. Using the developed DNA marker in combination with the ITS 2 nucleotide sequence, we monitored imported and commercially supplied medicinal products derived from *Cinnamomum* plants in markets in Korean, China, and Japan. The results revealed that most of the specimens monitored were derived from *C. cassia*.

**Electronic supplementary material:**

The online version of this article (doi:10.1007/s13258-016-0476-5) contains supplementary material, which is available to authorized users.

## Introduction

The genus *Cinnamomum*, which belongs to the family Lauraceae, contains approximately 250 known species (Leela 2008). Cinnamon, the dried bark of *Cinnamomum* species, such as a *Cinnamomum cassia*, *Cinnamomum verum* and other related species, is one of the most popular and important spices, and is also used in food flavorants, cosmetics, and medicines worldwide (Lai and Roy 2004; Mishra et al. 2009). Furthermore, different parts of a single species have different uses and effects. In traditional medicine, in particular, *Cinnamomum* plants are used in various forms, including Cinnamomi Ramulus (dried young branches of *C. cassia*), Cinnamomi Cortex (dried stem bark of *C. cassia*), and Cassiae Cortex Interior (dried stem bark, stripped off the thin cork layer of *C. cassia*) (Korea Institute of Oriental Medicine 2016).

*Cinnamomum* plants are generally distributed in the tropical and subtropical montane rain forests of Southern China, India, and Southeast Asia, and thus the Korean *Cinnamomum* market is completely dependent on imported products. In Sri Lanka and India, *C. verum* is cultivated as one of the most important spices, and is treated as true cinnamon that is widely used in the food and cosmetic industries (Swetha et al. 2014a, b). *C. cassia,* referred to as cassia cinnamon or Chinese cinnamon in Sri Lanka, is treated as an adulterant of *C. verum* (Thomas and Duethi 2001). However, according to the pharmacopoeias of Korea, China, Taiwan, and Japan, *C. cassia* is the only species of *Cinnamomum* officially permitted as the source of traditional medicines (Korea Institute of Oriental Medicine 2016). In the context of such imported products, the need has arisen for more reliable classification methods for *Cinnamomum* species, such that the quality of medicinal herbs, particularly cinnamon herbs, can be monitored. Traditionally, identification of *Cinnamomum* species has relied on expert botanical classification based on morphology or histological microscopy; however, identification based on morphological characteristics is difficult owing to the morphological similarities among species. Furthermore, it is virtually impossible to discriminate the species once the commodity loses its physical from; for example, when supplied as a powder.

Therefore, the aim of this study was to investigate the use of DNA analysis in discriminating among different *Cinnamomum* species based on the nucleotide sequences of their respective internal transcribed spacer (ITS) regions and the development of *C. cassia*-specific marker. We further sought to monitor the distribution of cinnamon herbs in the Korean market by using ITS sequences as DNA barcodes.

## Materials and methods

### Plant materials

#### Samples for identification

We collected 29 dried and/or fresh aerial parts, including leaf and bark specimens, from seven *Cinnamomum* species (*C. cassia, C. verum, C. burmanni,* *C. pauciflorum,* *C. iners,* *C. japonicum,* and *C. camphora*), which grow and/or are cultivated in the provinces of Vietnam, China, Indonesia, Sri Lanka, and Japan (Table 1). Specimens were dried at room temperature or frozen and stored at −70 °C. The authenticity of the specimens was verified by the Korea institute of oriental medicine (KIOM), the Department of Herbology in Wonkwang University, and Woosuk University.

**Table 1:** *Cinnamomum* plants used to determine the internal transcribed spacer (ITS) sequence

No.	Scientific name	Country of origin	Voucher no.	NCBI accession no.
1	*Cinnamomum cassia* (L.) J. Presl (=*Cinnamomum aromaticum*)	Vietnam	WKUCC01	KX766398
2			WKUCC02	
3			WKUCC03	
4			WKUCC04	
5		China	WKUCC05	
6			WKUCC06	
7			WKUCC07	
8		Indonesia	WKUCC16	
9			WKUCC19	
10		Sri Lanka	WKUCC22	
11	*Cinnamomum verum* J. Presl (=*Cinnamomum zeylanicum)*	Vietnam	WKUCC37	KX766399
12		Indonesia	WKUCC38	
13		China	WKUCC40	
14		Japan	WKUCC41	
15	*Cinnamomum burmanni* (Nees & T. Nees) Blume	Vietnam	WKUCC36	KX766400
16		Indonesia	WKUCC34	
17		China	WKUCC33	
18		Japan	WKUCC32	
19	*Cinnamomum pauciflorum* (=*Cinnamomum curvifolium* (Lam.) Nees)	China	WKUCC27	KX766401
20			WKUCC28	
21	*Cinnamomum iners* Reinw. ex Blume	China	WKUCC60	KX766402
22			WKUCC61	
23			WKUCC62	
24	*Cinnamomum japonicum* Sieb. (=*Cinnamomum tenuifolium* (Makino) Sugim.)	China	WKUCC63	KX766403
25			WKUCC64	
26		Korea	WKUCC65	
27	*Cinnamomum camphora* (L.) J. Presl	Japan	WKUCC66	KX766404
28			WKUCC67	
29		Korea	WKUCC68	

#### Monitoring samples

For the monitoring research, a total of 160 specimens used for medicinal purposes (70 Cinnamomi Cortex, 80 Cinnamomi Ramulus, and 10 Cassiae Cortex Interior) were obtained from commercial suppliers in Korean, Chinese, and Japanese markets (Table 3).

### Preparation of genomic DNA

Genomic DNA was extracted from each sample by using a NucleoSpin^®^ Plant II kit (Macherey–Nagel, Germany), according to the manufacturer’s instructions. Some of the samples required additional steps to improve the DNA quality. Phenolic compounds and polysaccharides were removed with 10 % cetyltrimethylammonium bromide and 0.7 M NaCl. After determination of the purity and concentration of the prepared genomic DNA using a NanoDrop DN-1000 Spectrophotometer (Thermo Scientific, Wilmington, DE, USA), the DNA was diluted and stored at −20 °C.

### Polymerase chain reaction (PCR) amplification

The ITS region of genomic DNA (including the 5.8S rRNA coding region of the nuclear DNA) was amplified from three samples of each specimen using the previously described universal primers ITS 1 (5′-TCCGTAGGTGAACCTGCGG-3′) and ITS 4 (5′-TCCTCCGCTTATTGATATGC-3′) (White et al. 1990), and nucleotide sequences were determined. PCR amplification was conducted in a 30-µl reaction volume containing 50 ng of genomic DNA, 1.2 pmol of primers, and 1 U Taq polymerase (ABgene, Epson, UK). Amplification consisted of pre-denaturation for 5 min at 95 °C, followed by 35 cycles of denaturation for 30 s at 95 °C, annealing for 30 s at 52 °C, and extension for 30 s at 72 °C, with a final extension for 5 min at 72 °C. The amplified products were separated on a 1.2 % agarose gel and visualized by staining with SafeView™ (Applied Biological Materials, Canada). PCR products extracted from the gel were purified using a LaboPass™ Gel Kit (Cosmo Genetech, Seoul, Korea).

### Analysis of DNA sequences

The determined nucleotide sequences were edited manually and aligned using ClustalW multiple sequence alignment in BioEdit v7.0.9 (http://mbio.ncsu.edu/BioEdit/bioedit.html). Genetic distances were calculated and dendrograms were constructed using neighbor-joining analysis of the data generated by DNADist in BioEdit. To study the relationship among *Cinnamomum* species, we used the nucleotide sequences of *Cinnamomum* species deposited in the National Center for Biotechnology Information (NCBI) GenBank database. Species of the genera *Sassafras* (AF272335.1), *Machilus* (AB260888.1), *Lindera* (AF272284.1 and AB470488.1), and *Litsea* (KP092872.1) were used as outgroups in the phylogenetic analyses. The ITS sequences of these taxa were obtained from the NCBI GenBank database.

### Amplification of DNA markers of *C. cassia*

DNA markers were amplified using a reaction mixture containing 1.2 pmol of the primer pair CC F1/CC R3, 1 U Taq polymerase (ABgene), and 50 ng of genomic DNA. Amplification consisted of pre-denaturation for 5 min at 95 °C, followed by 23 cycles of denaturation for 30 s at 95 °C, annealing for 20 s at 54.5 °C, and extension for 20 s at 72 °C, with a final extension for 5 min at 72 °C. To amplify an internal standard for evaluation of the PCR procedure, we used the primer pair ISF/ISR, which amplifies a 94-bp sequence. The amplified products were separated on a 1.2 % agarose gel and visualized by staining with SafeView™.

### Analysis of the ITS 2 sequence of monitored samples

The nucleotide sequence of the ITS 2 region was determined to confirm the monitoring results obtained using the *C. cassia* DNA marker. The previously described (White et al. 1990) universal primers ITS 3 (5′-GCATCGATGAAGAACGCAGC-3′) and ITS 4 (5′-TCCTCCGCTTATTGATATGC-3′) were used. PCR amplification for the 160 monitoring samples was conducted using same conditions employed for whole ITS region amplification. The determined nucleotide sequences were edited manually and aligned using ClustalW multiple sequence alignment in BioEdit v7.0.9 (http://mbio.ncsu.edu/BioEdit/bioedit.html).

## Results

### Analysis the ITS sequences

**Fig. 1:**
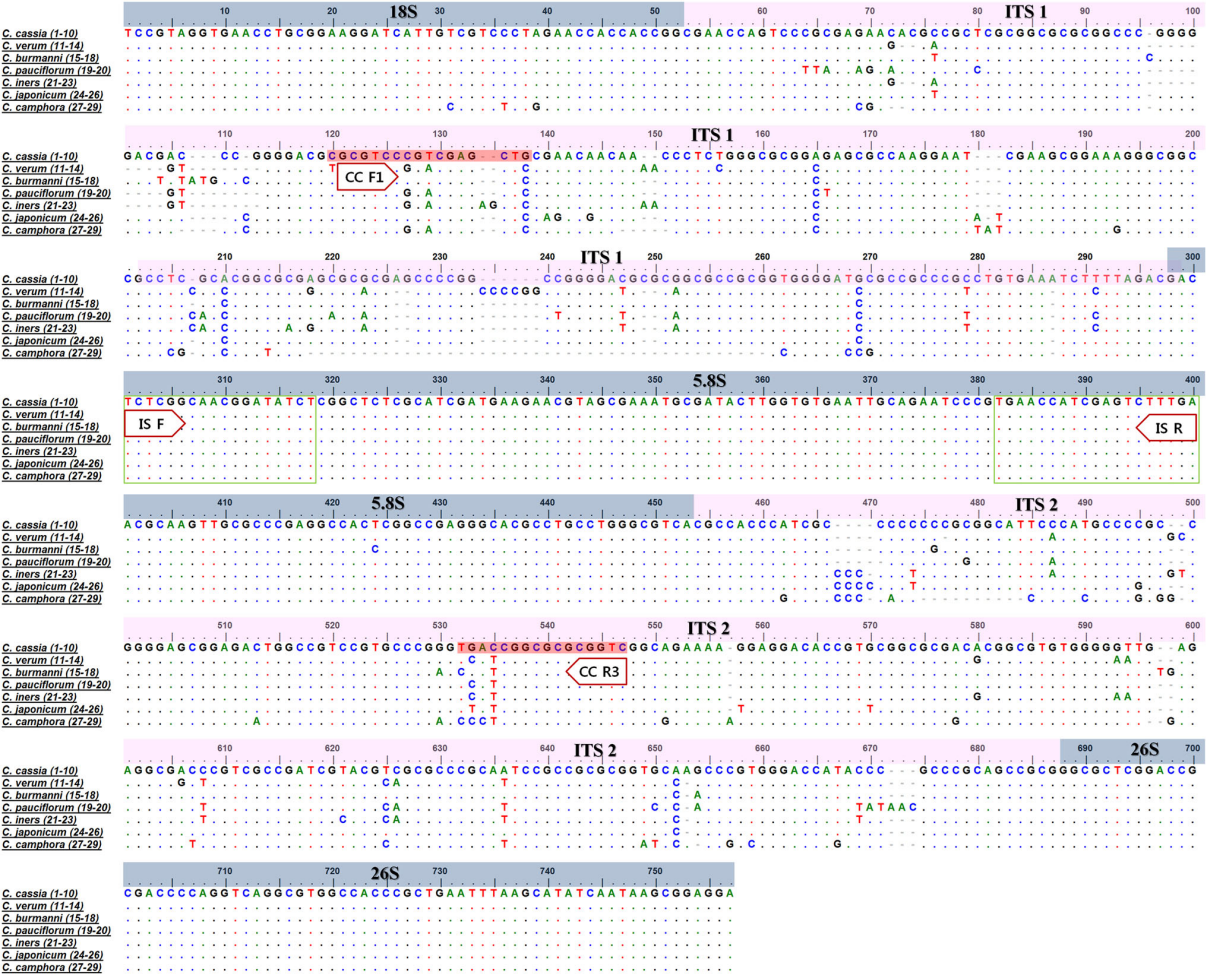
Multiple alignments of the nucleotide sequences of the internal transcribed spacer (ITS) region among *Cinnamomum* plants. The *dots* indicate the consensus nucleotides, and the *dashes* represent gaps. *Numbers* represent the sample numbers shown in Table 1. *Boxes* represent the primer pair developed in this study

The 29 specimens from seven species of *Cinnamomum* for which 680–729-bp nucleotide sequences of the ITS (including the 5.8 s region) region were determined are listed in Table 1. The determined ITS nucleotide sequences are presented in Fig. 1, and these have been deposited in the NCBI GenBank database: *C. cassia* (KX766398), *C. verum* (KX766399), *C. burmanni* (KX766400), *C. pauciflorum* (KX766401), *C. iners* (KX766402), *C. japonicum* (KX766403), and *C. camphora* (KX766404). As shown in Fig. 1, no differences were detected in the ITS nucleotide sequences among the intraspecific samples of the seven *Cinnamomum* species. However, differences in the ITS nucleotide sequences among the species were sufficient to enable discrimination of each species.

Homology of 85–97 % was detected among the ITS nucleotide sequences of the seven *Cinnamomum* species (Table 2). The results indicate that, compared with other *Cinnamomum* species, the ITS nucleotide sequence of *C. cassia* has highest homology with that of *C. burmannii* (97 %) and *C. japonicum* (96 %). The ITS nucleotide sequence of *C. verum* has 97 % homology with that of *C. iners*, whereas the sequence in *C. burmannii* shows 96 % homology with that of *C. japonicum*. The ITS nucleotide sequence of *C. camphora* shows an average 86 % homology with all the other six species, which was the lowest detected in the present study, whereas among the other six species, the average homology was greater than 92 %.

**Table 2:** Analysis of the homology of determined ITS nucleotide sequences among the seven *Cinnamomum* species listed in Table 1

(%)	*C. cassia*	*C. verum*	*C. burmanni*	*C. pauciflorum*	*C. iners*	*C. japonicum*	*C. camphora*
*C. cassia*	100						
*C. verum*	92	100					
*C. burmanni*	97	92	100				
*C. pauciflorum*	92	95	92	100			
*C. iners*	92	97	92	95	100		
*C. japonicum*	96	92	96	92	93	100	
*C. camphora*	87	85	87	86	86	88	100

### Analysis of phylogenetic relationships

**Fig. 2:**
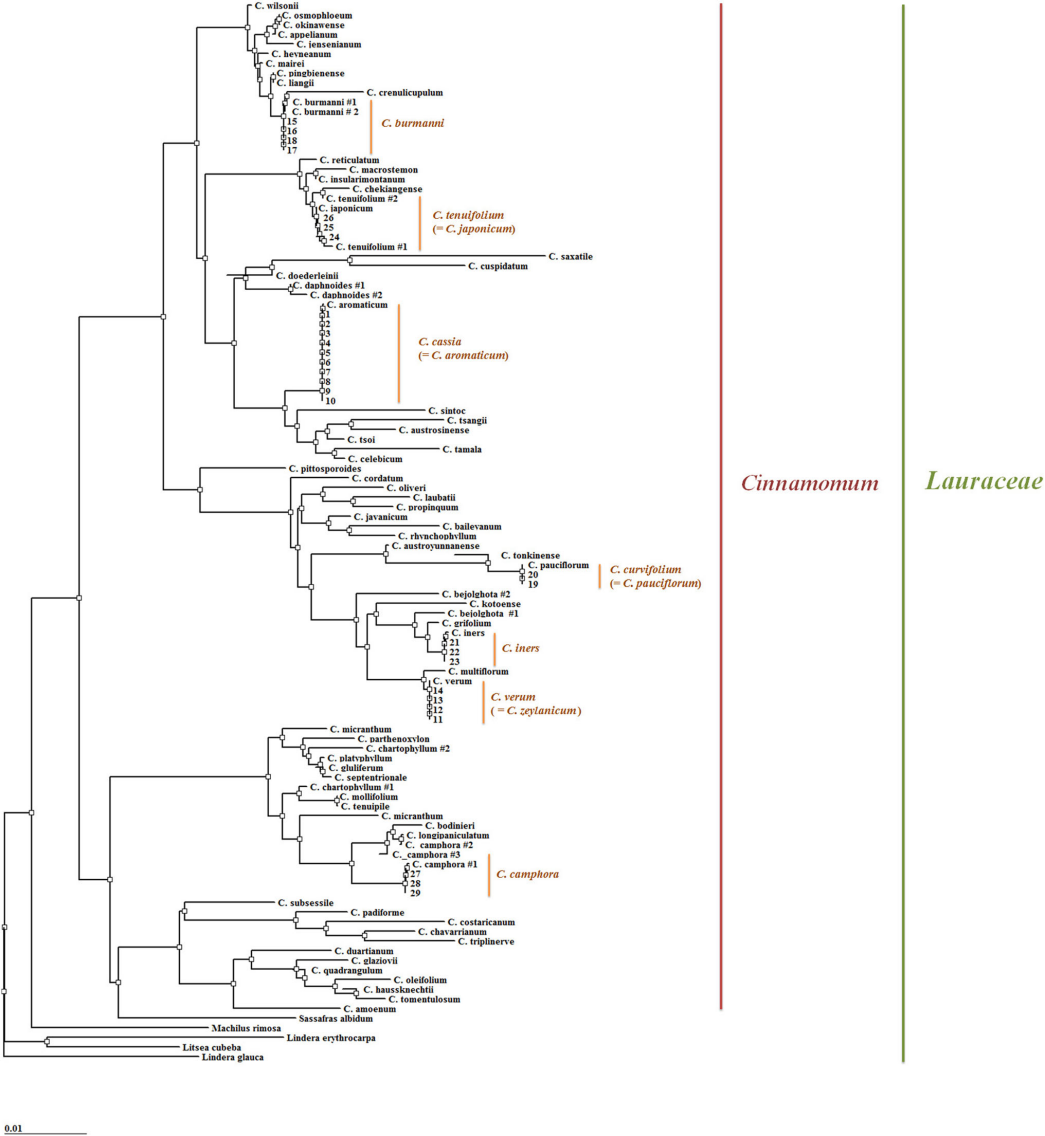
Dendrogram constructed based on the internal transcribed spacer (ITS) sequences presented in Fig. 1. As the outgroups, we used the ITS sequences of *Sassafras albidum* (Accession Number AF272335.1), *Machilus rimosa* (AB260888.1), *Litsea cubeha* (Number KP092872.1), *Lindera erythrocarpa* (Number AF272284.1) and *Lindera glauca* (Number AB470488.1) deposited in the NCBI GenBank

Phylogenetic relationships among the seven examined *Cinnamomum* species based on the determined ITS sequences were well resolved. As outgroups, we used the NCBI GenBank sequences of *Sassafras albidum* (Accession Number AF272335.1), *Machilus rimosa* (AB260888.1), *Litsea cubeha* (KP092872.1), *Lindera erythrocarpa* (AF272284.1), and *Lindera glauca* (AB470488.1), which, like the species of *Cinnamomum*, are classified in the family Lauraceae (Fig. 2). Furthermore, ITS nucleotide sequences of the genus *Cinnamomum* previously deposited in the NCBI GenBank database were used to overcome the disadvantage of the limited number of *Cinnamomum* species used for investigating phylogenetic relationships (Supplement 1). As represented in Fig. 2, each of the 29 specimens was separately grouped on the dendrogram according to the species of origin. On the basis of homology, *C. camphora* is located in a completely different cluster compared to the other six examined species. Specimens of *C. cassia* and *C. verum,* which are used under the common name “cinnamon,” are clustered in different groups. Similarly, *C. cassia*, *C. burmanni,* and *C. japonicum* are well divided into different groups, whereas *C. verum, C. iners*, and *C. pauciflorum* show a close phylogenetic relationship.

### DNA marker for *C. cassia* based on the discrepancy in the ITS sequences

**Fig. 3:**
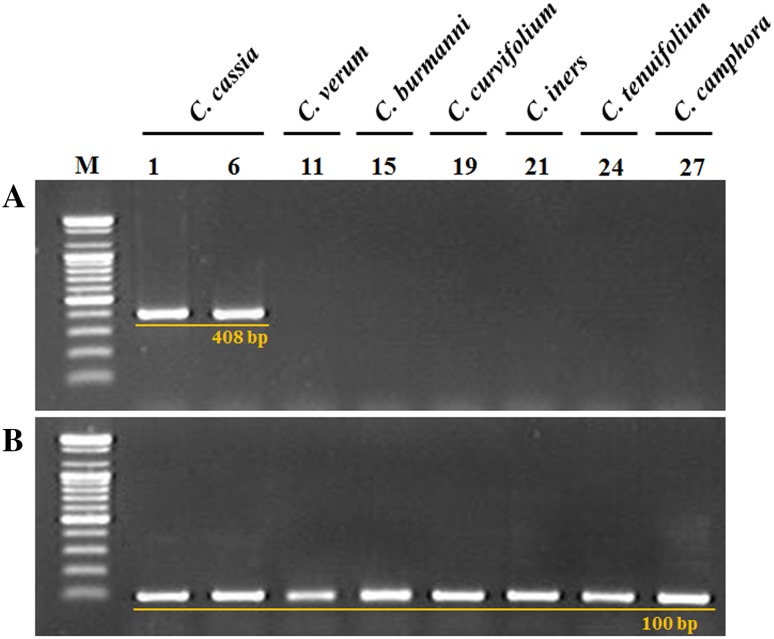
PCR products of the designed primer set, CC F1/CC R3 (**a**) and PCR products of the 5.8 s ribosomal RNA region, amplified with the ISF/ISR primer set (**b**) from six *Cinnamomum* species. *Lane numbers* are listed in Table 1. M: 100 bp ladder

On the basis of the findings presented in Figs. 1 and 2, we speculated that we could discriminate *C. cassia* from the other species of *Cinnamomum* examined in this study. To determine *C. cassia* more efficiently, we attempted to develop a DNA marker that could be used to discriminate *C. cassia* from other *Cinnamomum* species based on the discrepancy in the determined ITS sequences. We designed the primer CC F1 paired with CC R3 to amplify a 408-bp PCR product that appeared uniquely in the samples of *C. cassia* (Fig. 3). The sequences of the primer oligonucleotides are shown within the colored boxes in Fig. 1. The 5.8 s region was used to confirm the PCR amplification by using the ISF/ISR primer pair, shown in Figs. 1 and 3b.

### Monitoring traditional medicine derived from *C. cassia* in markets

As shown in Figs. 1, 2 and 3, we could efficiently discriminate *C. cassia* from other *Cinnamomum* species based on ITS sequences and the developed DNA marker. On the basis of these results, we attempted to monitor the traditional medicines derived from *Cinnamomum* plants in commercial markets. Three types of traditional medicine, Cinnamomi Cortex, Cinnamomi Ramulus, and Cassiae Cortex Interior, were collected from several markets in Korea, China, and Japan. To improve reliability, we also attempted to collect specimens from various cultivation regions. In total, we collected 160 specimens: 70 Cinnamomi Cortex, 80 Cinnamomi Ramulus, and 10 Cassiae Cotex Interior (Table 3).

**Table 3:** The identification of traditional herbal medicines derived from *Cinnamomum* plants, as determined by ITS nucleotide sequences

No.	Traditional medicine name	Country of origin	Location of commercial market	ITS2 barcode results
1–20	Cinnamomi Cortex	Vietnam	Korea	*C. cassia*
21–40	Cinnamomi Cortex	China		*C. cassia*
41–50	Cinnamomi Cortex	Indonesia		*C. cassia*
51–70	Cinnamomi Ramulus	Vietnam		*C. cassia*
71–90	Cinnamomi Ramulus	China		*C. cassia*
91–100	Cinnamomi Ramulus	Indonesia		*C. cassia*
101–103	Cinnamomi Ramulus	Sri Lanka		*C. cassia*
104–108	Cassiae Cortex Interior	Vietnam		*C. cassia*
109–113	Cassiae Cortex Interior	China		*C. cassia*
114–123	Cinnamomi Cortex	Vietnam	China	*C. cassia*
124–133	Cinnamomi Cortex	China		*C. cassia*
134–143	Cinnamomi Ramulus	Vietnam		*C. cassia*
144–153	Cinnamomi Ramulus	China		*C. cassia*
154–155	Cinnamomi Ramulus	Sri Lanka	Japan	*C. burmanni*
156–157	Cinnamomi Ramulus	Indonesia		*C. cassia*
158–160	Cinnamomi Ramulus	Vietnam		*C. cassia*

By using the *C. cassia*-specific marker to monitor the specimens, we were able to identify two specimens (numbers 154 and 155) as not being derived from *C. cassia,* whereas all the remaining samples of traditional medicine were confirmed to be derived from *C. cassia* (Supplement 2). In order to determine the possibility of contamination with other species and to verify the results obtained using the *C. cassia*-specific marker, we used the nucleotide sequence of the ITS 2 region. As shown in Fig. 1, the ITS 2 region facilitated sufficient discrimination among the *Cinnamomum* species (Supplement 3). According to the determination using the ITS 2 region, two samples (ID 154 and 155) were derived from *C. burmanni*, whereas the remainders were derived from *C. cassia* (Table 3). This result was consistent with the PCR identification using specific primers.

## Discussion

*Cinnamomum* is the largest genus in the family Lauraceae, comprising 250 species (Joy and Maridass 2008). Many species of *Cinnamomum* contain volatile oils, the most commercially important of which is cinnamon oil obtained from *C. verum, C. cassia*, and *C. camphora*. The major compounds in the stem bark of *Cinnamomum* plants are cinnamaldehyde (75 %) and camphor (56 %) (Senanayake et al. 1978). Cinnamon bark oil is employed mainly in the flavoring industry, but is also used for cosmetic and pharmaceutical purposes. In traditional medicine, other products derived from *Cinnamomum* plants, such as dried stem bark and young branches, are also used. In contrast to their medicinal use, there is no regulation regarding the species of *Cinnamomum* used for flavoring. According to the pharmacopoeias of Korea, China, Taiwan, and Japan, *C. cassia* is the only officially permitted *Cinnamomum* species that can be used as a source of traditional medicine (Korea Institute of Oriental Medicine 2016). However, the major of areas in which *Cinnamomum* plants are grown are distributed in tropical and subtropical Asia and Australia (Ho et al. 2015). Consequently, the *Cinnamomum* markets in many countries, including Korea, are completely dependent on imported products. Thus, the need has arisen for a reliable classification method for *Cinnamomum* species, so that the quality of medicinal herbs can be monitored.

Medicinal plants, including *Cinnamomum,* have long been utilized to treat diseases in traditional and modern medicine. Adulterants of traditional medicinal materials can originate from closely related species, or even species from other families (Li et al. 2011). Substitution and adulteration of medicinal plants can reduce the efficacy of the original drug, but in some cases, could make a drug lethal when substituted or contaminated with a toxic adulterant plant(s) (Techen et al. 2014). Therefore, authentication of medicinal plants is indispensable.

In this respect, DNA barcodes, which consist of short DNA sequences from a standard part of the genome, are useful tools for identifying species and discrimination at different taxonomic levels (Chen et al. 2009). Among the various loci used for the identification or discrimination of medicinal plants, the ITS region in the nuclear genome is one of the most useful loci.

As presented in Figs. 1 and 2, the discrepancy in the ITS sequences of *Cinnamomum* plants was the basis for developing a method for discriminating *C. cassia*, not only from the species shown in Table 1 but also from other species in the genus *Cinnamomum*. However, as previously mentioned, *Cinnamomum* is primarily found in tropical and subtropical Asia and Australia, and therefore the collection of specimens for examination has been limited. Several techniques used to identify *Cinnamomum* plants have been reported, including those based on RAPD (Joy and Maridass 2008; Sudmoon et al. 2014), chloroplast DNA (e.g., *trnL*-*F, trnL* intron, *matK, rbcL* and *trnH*-*psbA*) sequences (Sudmoon et al. 2014; Kojoma et al. 2002; Abeysinghe et al. 2009; Swetha et al. 2014a, b), and ITS nucleotide sequences (Ho et al. 2015; Abeysinghe et al. 2009). However, most of the research has focused on specimens from a limited number of species. Accordingly, in the present study, to overcome this disadvantage, we confirmed the discriminatory ability of the ITS sequences by using an extensive range of ITS sequences from the genus *Cinnamomum* deposited in the NCBI GenBank database. We therefore anticipate that analyzing the discrepancies in ITS sequences could be a valuable approach for discriminating *Cinnamomum* plants.

For more efficient discrimination of *C. cassia*, we designed the primer pair CC F1 and CC R3 based on the discrepancy in the determined ITS nucleotide sequences (Fig. 1). A 408-bp DNA marker was amplified solely in *C. cassia* specimens by the CC F1/CC R3 primer pair (Fig. 3). We then confirmed the nucleotide sequences of the amplified 408-bp products for accuracy of the *C. cassia*-specific DNA marker. On the basis of these results, we are convinced that the CC F1/CC R3 primer pair can discriminate *C. cassia*. The amplified product size is 408 bp, which is shorter than the normally studied DNA barcode regions, such as whole ITS regions, *rbcL,* and *matK.* It is very useful for application to dried and/or processed traditional medicines. Therefore, we anticipate that the developed *C. cassia* DNA marker will be used as an efficient method for monitoring and quality control of traditional medicines derived from *Cinnamomum* plants.

To conform this, we applied the newly developed *C. cassia* DNA marker to monitoring samples of Cinnamomi Ramulus, Cinnamomi Cortex, and Cassiae Cortex Interior. These traditional medicines are derived from *C. cassia*, and in Korean market in particular, they are entirely imported from other countries. To improve the reliability of the monitoring results, we collected specimens of Cinnamomi Ramulus, Cinnamomi Cortex, and Cassiae Cortex Interior from several different markets in Korea, and also collected samples from several markets in China and Japan, which also potentially could have been imported. On the basis of our results, most of the collected traditional medicine was identified as being derived from *C. cassia.* The exceptions being two (numbers 154 and 155) Cinnamomi Ramulus samples. We also used the ITS2 region to confirm the monitoring results. It has been shown to be valuable in identifying medicinal materials across 55 processed medicinal herbs belonging to 48 families (Chiou et al. 2007; Sun and Chen 2013). The success rate of identifying over 4800 plant species from 750 genera using ITS2 region is as high as 92.7 % and, indeed, in the case of the genus *Swartzia* in the family *Fabaceae*, the identification efficiency is nearly 100 % (Chen et al. 2010). The major advantage of the ITS 2 region for the differentiation of closely related species is the high inter-specific divergence and low intra-specific variation (Li et al. 2011). This sequence is also shorter than the entire ITS region, which makes it more appropriate for use in monitoring dried and/or processed traditional medicines. As shown in Table 3, the samples 154 and 155 differentiated in the present study were identified as *C. burmanni*. The remainder of the monitored samples were identified as *C. cassia,* which is consistent with the result obtained using the *C. cassia* DNA marker.

These results signify that the ITS nucleotide sequence, including the ITS2 region and the newly developed *C. cassia*-specific DNA marker, could be used as a reliable standard to identify and monitor traditional medicines originating from *Cinnamomum* plants. At present, the Cinnamomi Ramulus, Cinnamomi Cortex, and Cassiae Cortex Interior supplied to the Korean market for medicinal purposes originate from *C. cassia,* in accordance with pharmacopeia specifications. For further continuous monitoring and quality control of these traditional medicines, the *C. cassia*-specific marker developed in this study could be used as an efficient tool.

## Electronic supplementary material

Below is the link to the electronic supplementary material.

Supplementary material 1 (XLSX 13 kb)

Supplementary material 2 (JPEG 613 kb). PCR products amplified using the designed primer pair CC F1/CC R3 for monitoring specimens Lane numbers are listed in Table 3

Supplementary material 3 (JPEG 4794 kb). Multiple alignments result of the analysis of ITS 2 nucleotide sequences of monitored samples listed in Table 3
