# Supplementary figures and images for: Identification and monitoring of Korean medicines derived from Cinnamomum spp. by using ITS and DNA marker
Source: Genes Genomics. 2016 Oct 20;39(1):101–9. doi: 10.1007/s13258-016-0476-5 (PMC5196016; doi:10.1007/s13258-016-0476-5)

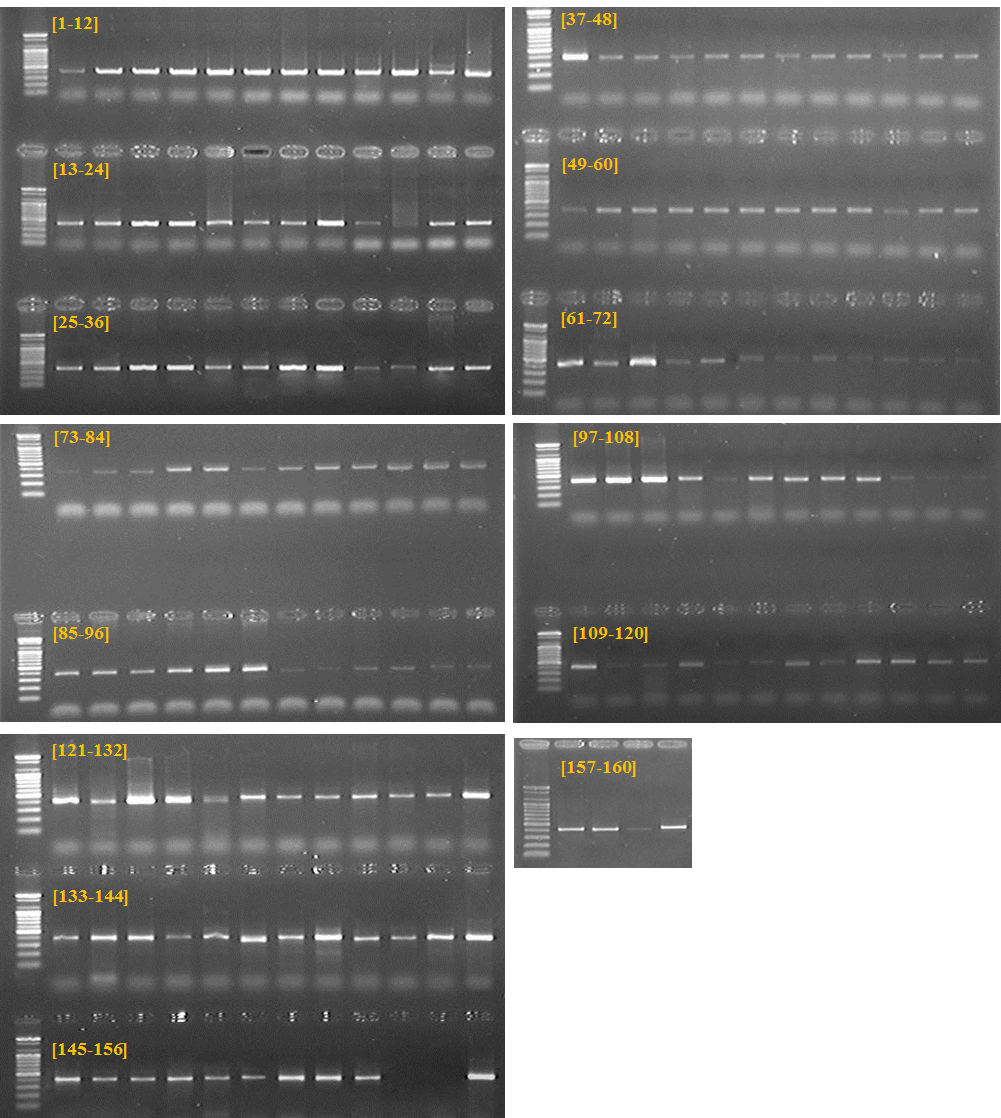

Supplement: Supplementary file 2 — Supplementary material 2 (JPEG 613 kb). PCR products amplified using the designed primer pair CC F1/CC R3 for monitoring specimens Lane numbers are listed in Table 3 [file 13258_2016_476_MOESM2_ESM.jpg]

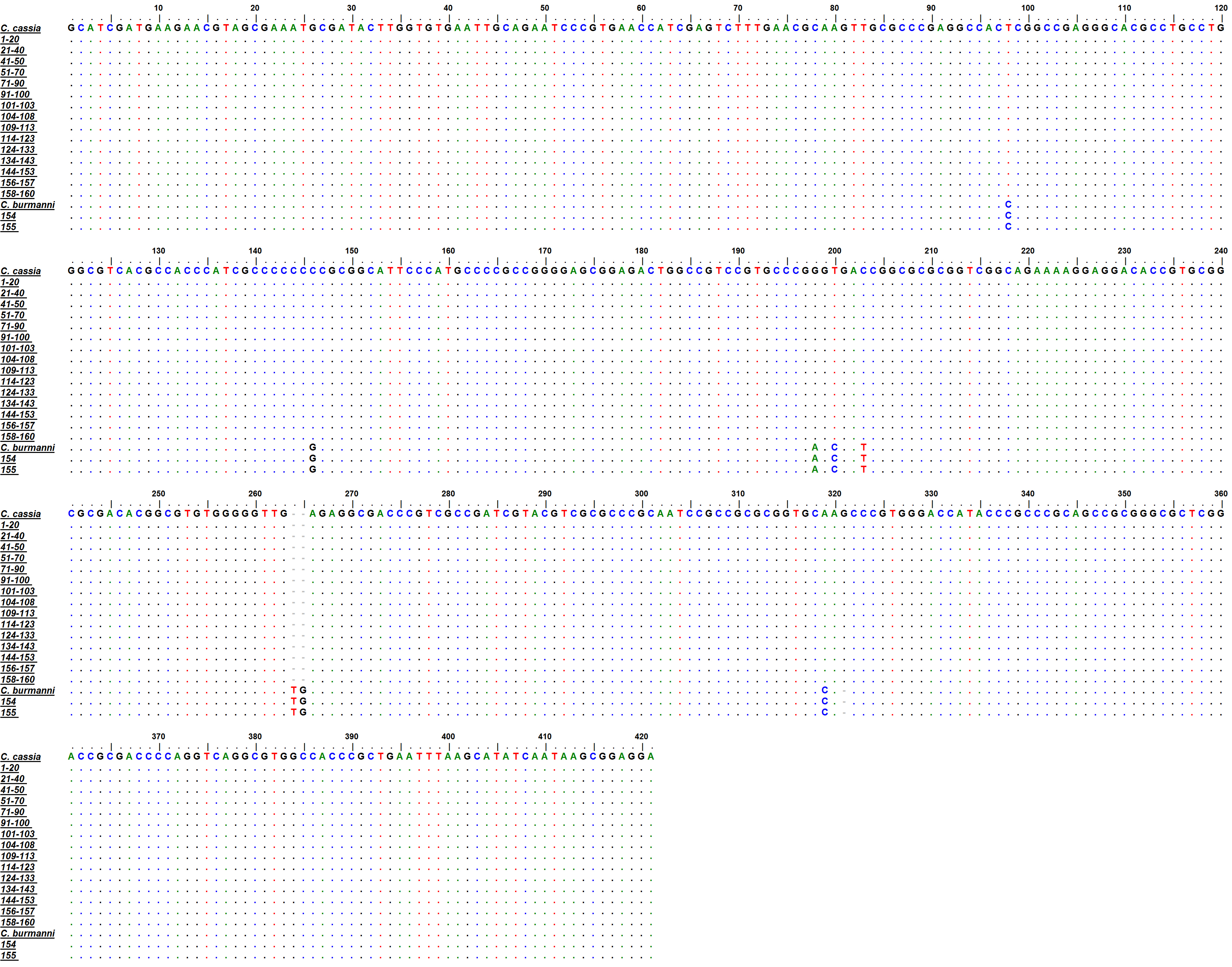

Supplement: Supplementary file 3 — Supplementary material 3 (JPEG 4794 kb). Multiple alignments result of the analysis of ITS 2 nucleotide sequences of monitored samples listed in Table 3 [file 13258_2016_476_MOESM3_ESM.jpg]
